# Calcium Imaging Reveals Fast Tuning Dynamics of Hippocampal Place Cells and CA1 Population Activity during Free Exploration Task in Mice

**DOI:** 10.3390/ijms23020638

**Published:** 2022-01-07

**Authors:** Vladimir P. Sotskov, Nikita A. Pospelov, Viktor V. Plusnin, Konstantin V. Anokhin

**Affiliations:** 1 Institute for Advanced Brain Studies, Lomonosov Moscow State University, 119991 Moscow, Russia; nik-pos@yandex.ru; 2 National Research Center “Kurchatov Institute”, 123098 Moscow, Russia; witkax@mail.ru; 3 Moscow Institute of Physics and Technology, 141700 Dolgoprudny, Russia; 4 P.K. Anokhin Institute of Normal Physiology RAS, 125315 Moscow, Russia

**Keywords:** Ca2+ indicators, calcium in vivo imaging, place cells, place fields, cognitive maps

## Abstract

Hippocampal place cells are a well-known object in neuroscience, but their place field formation in the first moments of navigating in a novel environment remains an ill-defined process. To address these dynamics, we performed in vivo imaging of neuronal activity in the CA1 field of the mouse hippocampus using genetically encoded green calcium indicators, including the novel NCaMP7 and FGCaMP7, designed specifically for in vivo calcium imaging. Mice were injected with a viral vector encoding calcium sensor, head-mounted with an NVista HD miniscope, and allowed to explore a completely novel environment (circular track surrounded by visual cues) without any reinforcement stimuli, in order to avoid potential interference from reward-related behavior. First, we calculated the average time required for each CA1 cell to acquire its place field. We found that 25% of CA1 place fields were formed at the first arrival in the corresponding place, while the average tuning latency for all place fields in a novel environment equaled 247 s. After 24 h, when the environment was familiar to the animals, place fields formed faster, independent of retention of cognitive maps during this session. No cumulation of selectivity score was observed between these two sessions. Using dimensionality reduction, we demonstrated that the population activity of rapidly tuned CA1 place cells allowed the reconstruction of the geometry of the navigated circular maze; the distribution of reconstruction error between the mice was consistent with the distribution of the average place field selectivity score in them. Our data thus show that neuronal activity recorded with genetically encoded calcium sensors revealed fast behavior-dependent plasticity in the mouse hippocampus, resulting in the rapid formation of place fields and population activity that allowed the reconstruction of the geometry of the navigated maze.

## 1. Introduction

It is well known that neurons in the CA1 field of the hippocampus form a representation (also referred to as a cognitive map) of a novel context, while animals explore a novel environment [1,2]. The long-term dynamics of such cognitive maps have been well explored in studies [3,4,5], revealing that the place code can be stable for weeks, though subserved by a drifting population of CA1 neurons. Moreover, it is known that multiple cognitive maps can coexist in the hippocampus in a stable manner and switch between different navigating sessions and even within the same navigating session [6,7].

However, the short-term dynamics of place field emergence and initial tuning are still ill-defined. In particular, it is unclear whether place fields are established at the first moment at which the animal arrives in a novel place, or whether several repeated visits are necessary for place cells to become tuned. A recent study of head-restrained mice in a virtual navigation task demonstrated “immediate” place cells that appear and fire in a stable manner from the first lap in a novel virtual environment [8]. However, it is unclear how rapidly the place codes emerge in real conditions of animal free navigation.

**Figure 1:**
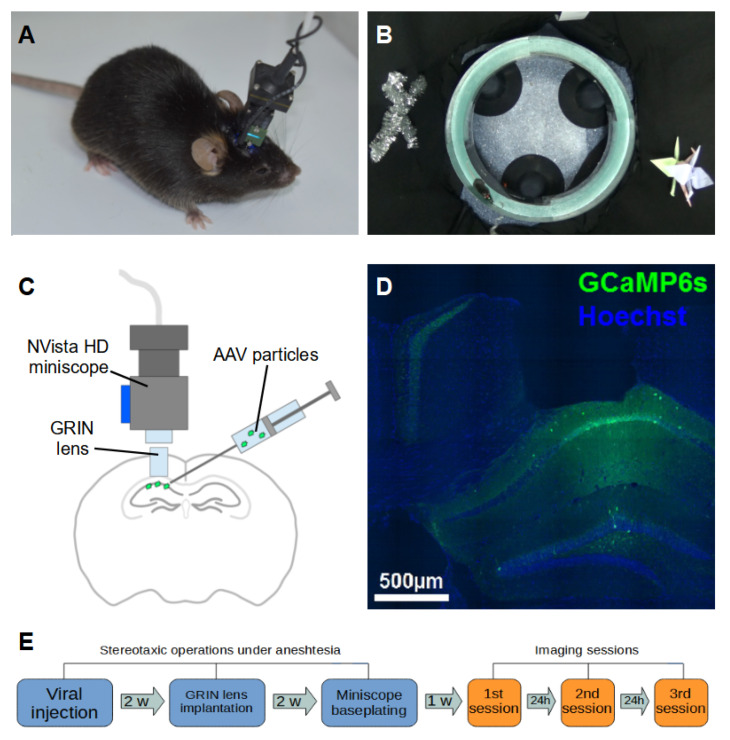
(**A**,**B**). Mouse with an attached NVista HD miniscope exploring the circular track. (**C**). A scheme of calcium sensor injection and GRIN lens implantation. (**D**). A coronal brain section with a footprint of a GRIN lens and calcium sensor expression. (**E**). The timeline of surgical preparations and imaging.

Many of the previous studies on place cell registration used rewarded approaches with pre-trained animals [7,9]. However, since goal-directed behavior may confound the factor of spatial navigation [10,11], we used a reward-free task where mice were allowed to explore a completely novel environment in the shape of an elevated circular track with proximal and distal visual cues (Figure 1).

To record place cell activity, we used head-mounted NVista HD miniscopes [12], which are capable of capturing calcium signals from hundreds of neurons in freely moving animals. To image cells, we used a set of genetically encoded calcium indicators: both conventional GCaMP6s and GCaMP7f, as well as novel ones, NCaMP7 and FGCaMP7. NCaMP7 is a new calcium indicator with enhanced brightness, containing a mNeonGreen fluorescent protein, while FGCaMP7 is a novel calcium sensor based on fungi calmodulin with lowered affinity to the intracellular environment and designed specifically for in vivo miniscopic calcium imaging [13,14]. In these papers, we described in detail their dynamic parameters, such as mean amplitude, and rise and decay times of typical calcium transients. Importantly, we showed that there is no notable difference in such parameters between these new calcium indicators and a conventional one, GCaMP6s. Moreover, the applicability of these sensors for the analysis of in vivo neural functions in awake mice was directly demonstrated by calcium imaging of hippocampal place cells.

Using these tools, we set out to investigate the dynamics of the initial place field formation in the mouse hippocampus, not only at the level of individual place cells, but also at the level of a whole imaged CA1 population. The activity of large populations of neurons is often well embraced by low-dimensional dynamics [15,16,17]. This makes it possible to describe computations performed by groups of cells using the dynamics of a small number of underlying “latent factors”, each of which corresponds to a separate pattern of neuronal coactivation. However, it is not possible to observe the latent factors directly, because they are often related to the initial variables in a non-obvious and non-linear way. In this paper, we utilize the tools from manifold learning to construct an underlying low-dimensional “neural manifold” from rapidly tuned place cell activity and to explore its representational power.

## 2. Materials and Methods

### 2.1. Animals and Surgical Procedures

Nine C57Bl/6J mice aged from 2 to 3 months at the beginning of the experiment were used for this study. All surgical protocols were described in detail in our previous papers [13,14,18,19]. First, a viral vector encoding one of the calcium sensors (GCaMP6s/GCaMP7f/NCaMP7/FGCaMP7) was delivered to the CA1 field of the hippocampus of the mice. Animals were anesthetized with a zoletil–xylazine mixture (40 and 5 mg/kg, respectively) and fixed in a stereotaxic holder (Stoelting Inc., Wood Dale, IL, USA). Then, a circular 2-mm-diameter craniotomy was made (Bregma: −1.9 mm AP, −1.4 mm ML), and 500 nL of AAV viral particles (AAV-DJ-CAG-GCaMP6s, AAV-DJ-CAG-GCaMP7f, AAV-DJ-CAG-NCaMP7 or AAV-DJ-CAG-FGCaMP7) was injected to a depth of 1.25 mm from the brain surface. Injections were performed through a glass micropipette with a 50 µm tip diameter (Drummond Scientific Comp., Broomall, PA, USA) by UltraMicroPump with a Micro4 Controller (WPI Inc., Sarasota, FL, USA) at a rate of 100 nL/min. After the injection, all exposed surfaces of the brain tissue were sealed with KWIK-SIL silicone adhesive (WPI Inc., Sarasota, FL, USA). Two weeks later, the animals were anesthetized and fixed in the stereotaxis again, the silicone cap was removed, and the dura mater was perforated and gently removed from the craniotomy site. Then, a column of cortex tissue superficial to the hippocampus was gently aspirated by a blunt needle tip connected to a vacuum source and the hippocampus was exposed and washed with sterile saline. After this, a 1.0-mm-diameter GRIN lens probe (Inscopix Inc., Palo Alto, CA, USA) was lowered slowly to a depth of 1.1 mm while constantly washing the craniotomy site with sterile saline. Next, all the exposed brain tissue was sealed with KWIK-SIL, and the lens probe was fixed to the skull surface with dental acrylic (Stoelting Inc., Wood Dale, IL, USA). After another two weeks, the animals were checked for fluorescent calcium signal under light anesthesia ($\frac{1}{2}$ of the dose described above). The mice were fixed in the stereotaxis, and an NVista HD miniature microscope (Inscopix Inc., Palo Alto, CA, USA) was lowered upon the GRIN lens probe and the optimal field of view was chosen. Then, a baseplate for chronic imaging was affixed to the skull surface with dental acrylic.

### 2.2. Miniscope Imaging in Freely Behaving Mice

**Figure A1:**
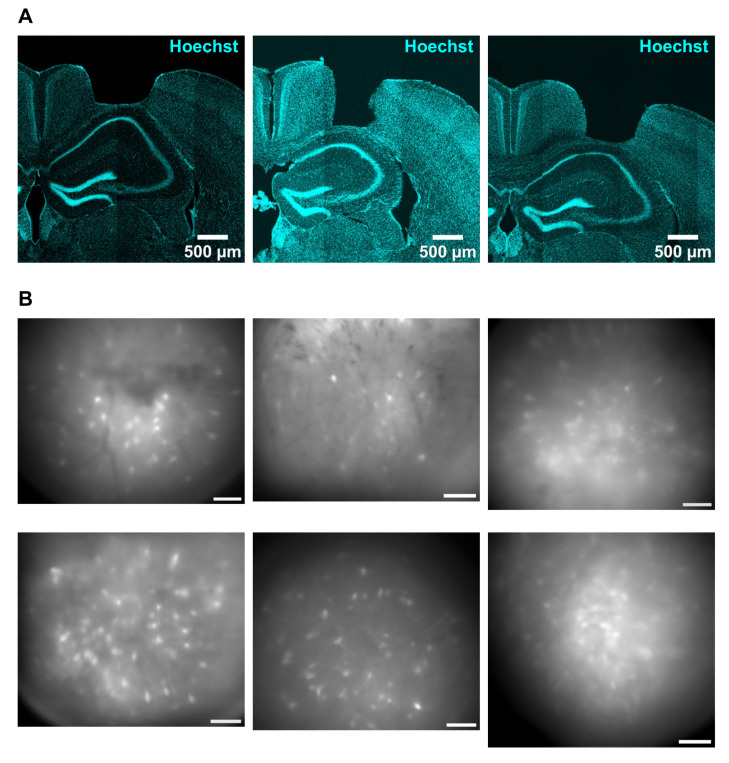
(**A**) Samples of histology sections indicating GRIN lens implantation locations stained with Hoechst. (**B**) Samples of raw NVista HD video screenshots. Scale bars, 100 µm.

Finally, after a one-week recovery period, awake mice with an attached NVista HD miniscope were placed for 15 min into a custom-made circular O-shaped track (50 cm diameter, 5 cm width, with 5 cm height borders) with proximal (different border material) and distal (placed on a surrounding curtain 20 cm apart from the track) visual cues. Mice were allowed to explore the environment in arbitrary directions and were not forced to move. The imaging session was repeated for 8 of 9 mice on the next day after 24 h and for 3 mice on the third day after 48 h from the first imaging session. The neural activity was recorded at 20 frames per second at resolution 1440 × 1080 px with an NVista HD miniscope. Screenshots and video samples of raw calcium signal can be seen in Figure A1 of Appendix A and in Supplementary Videos S1–S3.The video of mouse behavior was captured with a Sony HDR CX-405 (Sony Corp., Tokyo, Japan) camera at 25 frames per second.

At the end of the experiments, animals were perfused transcardially with 1% paraformaldehyde in 0.1 mM CaCl ${}_{2}$, and then brains were extracted and postfixed for 24 h in the same solution. Thin (50 µm) floating sections were prepared with a Leica 1200VT (Leica Microsystems GmbH, Wetzlar, Germany) vibratome, stained with Hoechst dye (Hoechst AG, Frankfurt, Germany) and imaged with an Olympus FluoView 1000 (Olympus Corp., Tokyo, Japan) confocal microscope with a UMPlanFLN 10× NA 0.30 W objective. All sections were inspected and checked for consistency of calcium sensor expression site and GRIN lens implantation locations to the field CA1 of the hippocampus. Samples of such sections can be seen in Figure 1D and in Figure A1 of Appendix A.

### 2.3. Neural and Behavioral Data Processing

**Figure 2:**
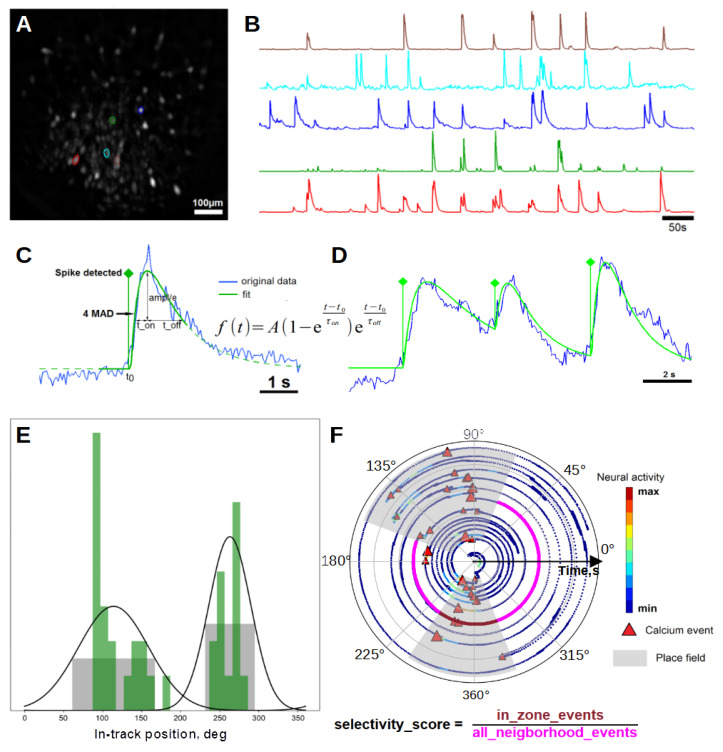
(**A**,**B**) Selected neuron locations (**A**) and their traces (**B**) extracted by MIN1PIPE routine. (**C**) Scheme of spike detection. Whenever the trace reaches the threshold value, the fitting procedure starts in a range, denoted by the solid green line. Fitting curve *f(t)* is set to a composition of rise and decay exponential factors and spiking amplitude *A*, where t_0_, t_on_ and t_off_ are fitting parameters. (**D**) Scheme of multiple spike detection. In case of tolerable goodness of fit (not less than 0.8), the event is scored, and the fitting curve is subtracted from the original trace, allowing next peaks to be scored. (**E**) Spatial event distribution for putative place field detection. Solid black line denotes Gaussian mixture fit of the distribution and grey zones are putative place fields. (**F**) An example of a detected place field based on the distribution on the left. Trajectory of the mouse is unfolded in the axial direction for better readability and colored with respect to raw activity of the correspondent place cell. To check the consistency of putative place field, we calculated selectivity score each time the animal attended place field zone (in the depicted case, selectivity score equals 1).

Image processing was performed with the NoRMCorre [20] and MIN1PIPE [21] pipelines and custom MATLAB and Python scripts. First, all movies were downsampled spatially by a factor 2 to increase the computation speed. Then, the NoRMCorre routine was applied to spatially align movies and to correct motion artifacts. Next, the MIN1PIPE routine was applied to corrected movies, and locations and activity traces of putative cell units were extracted and manually inspected (Figure 2A,B). Then, significant calcium events were detected in the activity traces.

Detection of calcium events was performed with a custom routine, which was described in our previous work [18]. First, a threshold of 4 or 5 median absolute deviations (MADs) was applied to extracted neural traces for animals injected with slow (GCaMP6s, NCaMP7 and FGCaMP7) or fast calcium sensors (GCaMP7f), respectively. Then, neighborhoods of each upward threshold crossing were fitted with a typical calcium event model function with fast rise and slow decay (Figure 2C). This model utilizes precise spiking time t_0_, rise time t_on_, decay time t_off_ and spiking amplitude *A* as the parameters to be optimized. The lower limit of t_off_ was set to 200 ms for mice injected with the GCaMP7f indicator and 500 ms for the other ones. In case of acceptable fit (goodness of fit ≥0.8), a calcium event was scored and the fit was locally subtracted from the original trace in order to let subsequent events be fitted and scored (Figure 2D).

**Figure A2:**
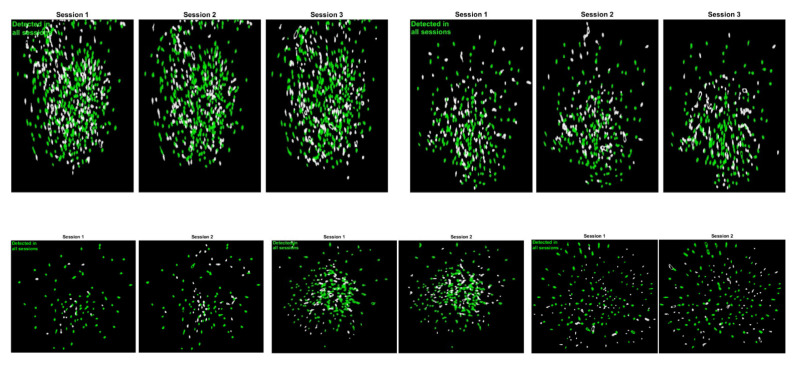
Samples of cell matching between sessions. Matched population is marked in green.

Data for each session were processed separately; matching of cells and traces across sessions was performed with the CellReg routine [22] with default parameters (maximum angle of 30 degrees, maximum translation of 14 microns, registration threshold P_same of 0.5). Exact amounts of matched cells across sessions and their contours can be seen in Table A2 and in Figure A2 of Appendix A. Positions of animals were extracted from behavioral video recording with the open-source Bonsai visual programming media [23]. All obtained time series were synchronized and aligned to the beginning of the imaging session.

### 2.4. Place Cell Detection

Given some uncertainty that exists in the procedure of place cell detection [24] and that the commonly used approach based on spatial information [25] requires a high running speed of animals and does not consider the repeatability of place cell firing each time the animal visits the place field, we used a conservative approach, where we checked both the spatial and temporal persistence of place cell firing. The entire track space was divided into 20 sectorial bins sized 5 × 7.5 cm. For each cell, an overall number of calcium events was calculated for each bin and the distribution of calcium events was smoothed with a Gaussian kernel (sigma = 1.25), normalized and thresholded by a value of 0.5. Then, centroids of all subthreshold peaks were considered putative place field centers. Width of place fields was evaluated as width of subthreshold peaks + 1 bin from each side. Place fields wider than a half of the track were excluded from further analysis. Next, for each place field, we checked whether the correspondent cell fired in all epochs when the animal attended a given place field. For this purpose, for each attendance of a given place field, we calculated the selectivity score as a ratio of the number of events within the attendance epoch over the total number of events within this epoch and adjacent epochs between visits (Figure 2F). For each place field, we smoothed the selectivity score sequence with a Gaussian filter (sigma = 1). Only epochs of attendance with smoothed selectivity scores more than 0.5 were considered relevant; place cells without at least three subsequent relevant epochs were discarded from the analysis. The tuning latency for each place field was calculated as the number of epochs (or as time in seconds) before the beginning of the first sequence of three or more relevant epochs. We considered that the cognitive map retained between sessions in case of significant similarity of the distribution of place field location shifts between sessions to a normal distribution around zero (*p* < 0.05, Chi-square test).

### 2.5. Dimensionality Reduction

In this paper, we used the Laplacian eigenmaps method, which constructs a discrete approximation to a continuous low-dimensional representation that naturally arises from the geometry of the manifold [26]. Before dimensionality reduction, the calcium data for each animal were presented in the form of the matrix $D_{N\times T}$, where *N* is the number of selected cells and *T* is the total duration of recording. Therefore, $D_{ij}$ stands for the calcium activity of the neuron *i* at the timeframe *j*. Only neurons with 5 or more calcium events during the recording were considered.

We built a similarity graph *G* based on the data in the original high-dimensional space. Each vector $V_{t}$ was considered a point in $R^{N}$. Thus, each column of the matrix $D_{N\times T}$ was treated as a single *N*-dimensional vector of neural activity at a certain timeframe. Hence, we obtained *T* multidimensional vectors in $R^{N}$, representing neural activity in different moments in time: $\left\{V_{t}\right\},t\in \left[0,T\right]$. To simplify the graph construction procedure and reduce the computation time, we applied mean filtering to the initial time series of calcium activity with window size w=2,4,8. We made sure that the choice of the window size did not qualitatively affect our results. This is because the calcium signal hardly changes during the time corresponding to the window sizes of 40, 80, or 160 ms, respectively. All results here were obtained for window size w=8. Thus, the effective signal length and number of nodes in G was $T_{eff}=T/w$.

For each of the $T_{eff}$ similarity graph nodes, exactly *k* nearest neighbors were calculated using Euclidean distance. The number of nearest neighbors *k* was the only free parameter in our dimensionality reduction procedure. It tended to be chosen as small as possible (however, its value should have ensured the integrity of the resulting graph). Keeping *k* small is motivated by the local linearity assumption: if *k* becomes sufficiently large, the Euclidean distance may not reflect the proximity relations between data points because of the possible nonlinear curvature of the underlying manifold. The situation is also complicated by the dimensionality curse in the initial space [27].

The resulting graph adjacency matrix was explicitly made symmetric to preclude directed edge formation. This means that the “nearest neighbor” relation is made mutual: if some node *i* is connected to a node *j*, the reverse is also true. It should be noted that, due to the symmetrization procedure, the number of nearest neighbors *k* sets only the minimal number of edges for a given node, but not a precise one. The similarity graph was ensured to be connected. If it had more than one connected component after the construction procedure, the largest one was taken (if the total share of discarded points did not exceed 5%; otherwise, dimensionality reduction for a given *k* was considered unsuccessful). The time moments corresponding to the excluded points were not considered further in the analysis.

Once a similarity graph was constructed, it was presented in the form of an adjacency matrix $A=\{a_{ij}\}$ with matrix elements $a_{ij}=a_{ji}$ taking non-negative values. The absence of self-loops implies the vanishing of the diagonal elements: $a_{ii}=0$. The matrix elements were considered binary: $a_{ij}=1$, if the nodes *i* and j≠i are connected, and $a_{ij}=0$ otherwise. The next step was constructing a discrete Laplacian—a symmetric, positive semidefinite matrix that can be considered a diffusion operator on the graph *G*. The spectral decomposition of the graph Laplacian matrix can be used to optimally embed the graph in a low-dimensional space [26]. According to the Laplacian eigenmaps algorithm, we considered the following generalized eigenvalue problem:

(1) Lv=λDv

Here, *D* is the degree matrix of the network, whose elements are defined as $d_{ij}=deg\left(n_{i}\right)$ if *i* = *j* and $d_{ij}=0$ otherwise, where $deg(n_{i})$ is the degree of the node *i*: $deg\left(n_{i}\right)=\sum_{j}^{}a_{ij}$. *L* stands for the graph Laplacian matrix, which is defined as

(2) L=D−A

The algorithm utilizes first m+1 solutions $\left\{f_{i}\right\},i\in \left[0,m\right]$ of the generalized eigenvalue problem (1) (ordered by the associated eigenvalues $\left\{\lambda_{i}\right\}$ in the ascending order) to construct an optimal embedding of the graph in $R^{m}$. To be precise, the *k*-th component of an eigenvector $u_{i}$ defines an *i*-th coordinate of a low-dimensional embedding for a data point $v_{k}\in R^{N}$. Since the dimensionality of $u_{i}$ is equal to the number of nodes in the graph, the first *m* non-trivial eigenvectors are enough to construct a *m*-dimensional embedding for each data point.

The first eigenvector $v_{0}$ corresponding to $\lambda_{0}=0$ was left out because its components were constant. It is known that a Laplacian matrix of a graph with *c* connected components has *c* zero eigenvalues [28]. However, we restricted ourselves to the case of connected graphs, which was ensured by the construction procedure. Hereinafter, we assume that, for our graph, the problem (1) has a single zero eigenvalue.

To measure the reconstruction error of the track geometry, we calculated the residual variance (RV) [29] between real mouse coordinates and points in the latent space. The residual variance was calculated as $RV=1-\rho^{2}\left(D_{h},D_{l}\right)$, where ρ defines the Pearson correlation, and elements of $T_{eff}\times T_{eff}$ matrices $D_{h}$ and $D_{l}$ denote the pairwise Euclidean distances calculated over the original trajectory points over the low-dimensional embedding points, respectively.

## 3. Results

In the previous studies [13,14,18,30], we developed a non-rewarded paradigm, where mice with a head-mounted NVista HD miniscope explored a custom-made O-shaped circular track surrounded by curtains with distinctive visual cues (Figure 1B). Mice demonstrated vigorous exploratory behavior, making on average 19 laps across the track during a 15 min imaging session. It should be noted that since mice were able to arbitrarily change the moving direction, the number of laps varied significantly from session to session, and some of the laps were not full.

The mice were transfected with AAV vectors carrying different calcium indicators (namely GCaMP6s, GCaMP7f, NCaMP7 and FGCaMP7; detailed information can be seen in Table A1 of Appendix A). All mice underwent identical surgical, imaging and behavioral protocols. Mice explored the track at one, two or three consequent sessions, and the first time the context was absolutely novel for them. We isolated neuron locations, calcium traces and detected place-selective cells. For this, we selected candidate cells by the presence of distinct peaks in the overall (across entire session time) spatial distribution of calcium events of a given cell, and then checked if this cell fired or not each time the animal entered its putative place field. Only candidate cells with stable firing statistics throughout the session were considered place-selective (for more details, see Methods). We allowed each cell to have multiple place fields; however, the majority (85% on average) of place cells had single place fields.

**Figure 3:**
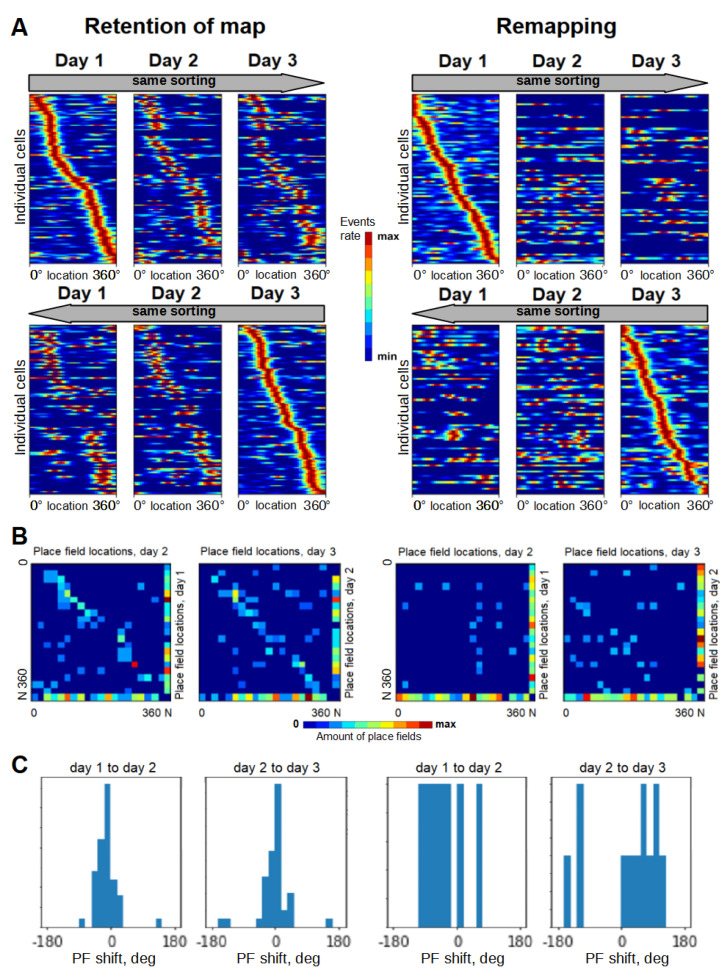
(**A**) Distribution of place fields across days in a case of retained representation (left) and in a case of remapping (right). Each line in a block corresponds to overall activation rate across the track space of each individual place cell in three consequent sessions. Upper row: cells sorted by their peak firing locations on the 1st day. Lower row: cells sorted by their peak firing locations on the 3rd day. (**B**) Heat maps of place cells that changed their place field locations between days. Multiple place fields of the same place cell are scored as fractions. N, not a place cell. Between-day transitions with the retention of the map result in heat maps with distinct diagonals, while remapping transitions do not. (**C**) Distribution of place field shifts for single-place-field place cells (between 1st and 2nd days and between 2nd and 3rd days). Between-day transitions with the retention of the map show sharp peaks in such distributions while remapping transitions show uniform distributions.

On the first day, all animals demonstrated a uniform distribution of place fields across the track, without any distinct fluctuations in the vicinity of visual cues or other locations in the track. We matched cell activity across days and monitored the place selectivity of the same cell on all days. It turned out that, on the second day, 3 of 8 imaged mice had cognitive maps similar to the first day (Figure 3). In the other five mice, cognitive maps were not preserved, which may have been partially due to cells that were not active on the first day.

### 3.1. Selectivity Score and Tuning Latency

**Figure 4:**
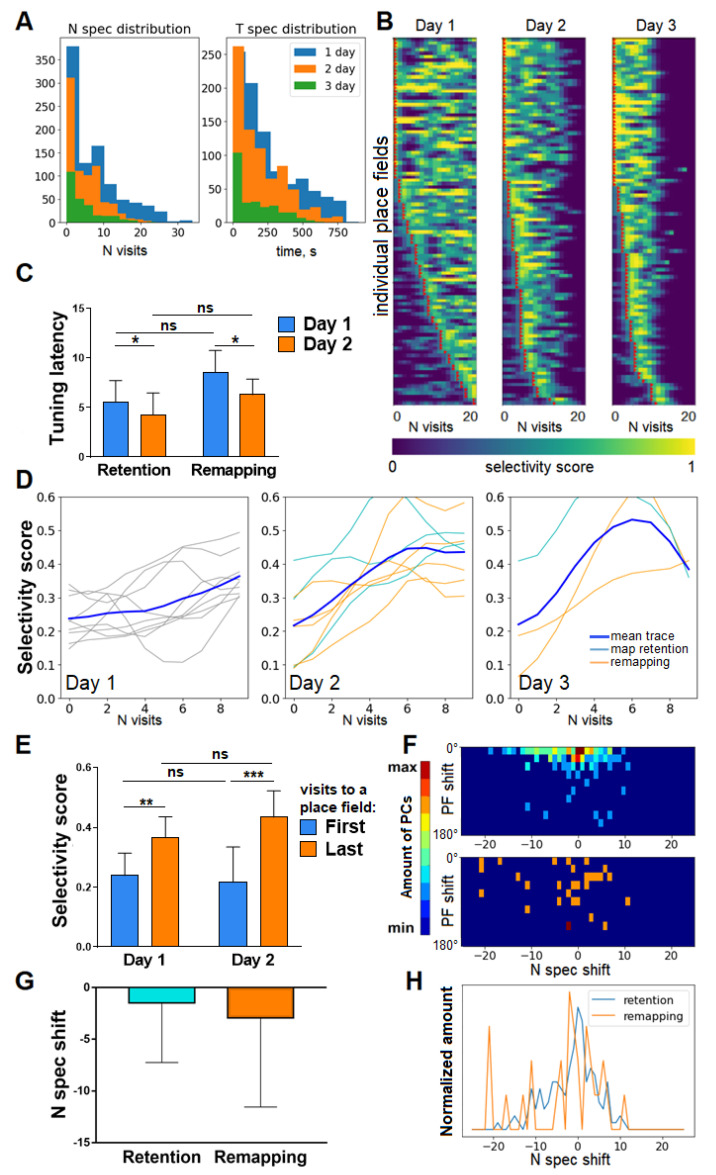
(**A**). Distribution of tuning latency both in the number-of-visits domain and in the time domain. (**B**). Sample of selectivity score distribution in one individual mouse on three consequent days (place fields are sorted independently for each session). Red triangles denote the number of visits where the specialization occurred (n_spec). (**C**) Tuning latency significantly decreases on the second day, while no difference is observed between retained-map and remapped mice. Two-way ANOVA, * *p* = 0.0488 factor day, ns—not significant (*p* > 0.05). (**D**) Evolution of mean selectivity score across days in all mice. Mean scores for each animal are represented in thin lines. (**E**) Mean selectivity score significantly rises within the first and the second day from the first to the last visit to a place field, while no difference is observed between starting or ending selectivity score on the 1st versus that on the 2nd day. Two-way ANOVA and post hoc Bonferroni test, ** *p* = 0.0261, *** *p* = 0.0006, ns—*p* > 0.05. (**F**) Scatter plot of individual cell variance between 2nd and 1st days in n_spec (n_spec shift) versus place field shift for retained-map mice (above) and remapping mice (below). Only one-field place cells were taken into account. (**G**) Shifts in n_spec do not differ significantly between retained-map and remapped mice. Unpaired Student’s t-test, *p* = 0.2544. (**H**) Distribution of n_spec shifts across all one-field place cells for mice with the retention of the map and for mice with remapping.

To assess the spatial selectivity of place cells to their fields, we used the selectivity score: for each place field, it was calculated whenever the animal attended the place field as the ratio of the number of in-field calcium events of the place cell over the total number of calcium events across the current lap (which may be not full). A selectivity score of 1 corresponded to a cell that fired exclusively in its place field and a score of 0 took place when the place cell did not fire at its place field during this visit. The time and the number of attendances when the smoothed selectivity score hits the threshold value of 0.5 are considered as the tuning latency or time of specialization. Since each mouse had its own trajectory and some mice explored the track faster than others, the number of visits appeared to be a more universal parameter for the estimation of the tuning dynamics of individual place cells rather than time itself. The distributions of tuning latency both in the time and number-of-visits domains are shown in Figure 4A. On the first day, a notable percentage (25.1%) of place fields were established at the very first attendance, while an average place field was formed at the 7th attendance. In the time domain, 23.1% of fields appeared within the first minute in the environment, while the average tuning latency equaled 247 s. On the second and third days, the average tuning latency of place fields decreased to values of 193 and 159 s, respectively, values that correspond to the 5th attendance of the place field. The improvement of tuning latency nominated in visits on the 2nd day was found significant (Figure 4C).

### 3.2. Selectivity Score Dynamics within Session and across Days

Regarding the selectivity score itself, its evolution was distributed in a similar manner (Figure 4B) across all animals, characterized by strong decay of the rate of cells with longer tuning latency. The mean selectivity score significantly increased from the first to the last attendance on each day of the experiment, but the between-days difference in selectivity score at the first and the last attendance appeared to be not significant (Figure 4D,E). Moreover, no significant difference in selectivity score improvement was observed on the 2nd day between mice with a retained cognitive map versus mice in which the map was not retained. Since we did not observe any cumulation of average selectivity score between days, we searched for it at the level of individual place cells. Importantly, the improvement in tuning latency appeared to be independent of the place field shift between sessions, i.e., cells that preserved their place field did not improve their tuning latency better than cells whose place field shifted (Figure 4F–H).

Taken together, these data suggest that the selectivity of place cell firing rose faster with each new day of the experiment, but without any significant cumulation. The retaining or remapping of the spatial representation in mice does not correlate with significantly higher selectivity scores or faster tuning dynamics. However, since these results are based only on individual place cell firing statistics, and since not only place cells can contribute to spatial coding [4,31], we performed a population analysis to confirm our results.

### 3.3. Nonlinear Dimensionality Reduction Reveals Track Geometry from Multidimensional Place Cell Activity

**Figure 5:**
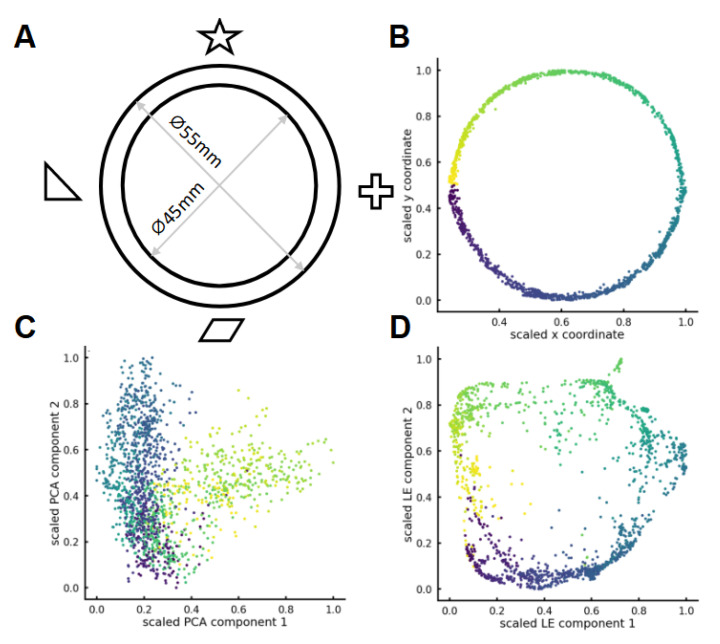
(**A**) Round track with visual cues, (**B**) pointwise track representation, (**C**) first two axes of PCA embedding, (**D**) first two axes of LE embedding (eigenvectors of the graph Laplacian).

We performed a populational analysis of the neural data of the first six mice with a sufficient (>200) number of detected cells on the 1st day and reduced the dimensionality of the data with Laplacian eigenmaps (see Methods). The first two axes of the low-dimensional space coincided with the coordinates of the mouse in the physical environment that it was exploring (with the accuracy of rotation by a fixed angle: Figure 5A,B). It is important to note that the algorithm did not receive any information about the real position of the mouse as an input. This result could not be reproduced with PCA, indicating the nonlinear nature of the problem (Figure 5C). The best result was achieved using low-energy Laplacian eigenmodes of the similarity graph of neuronal activity vectors (Figure 5D).

### 3.4. Dependence of Geometry Coding Quality on Population Size

**Figure 6:**
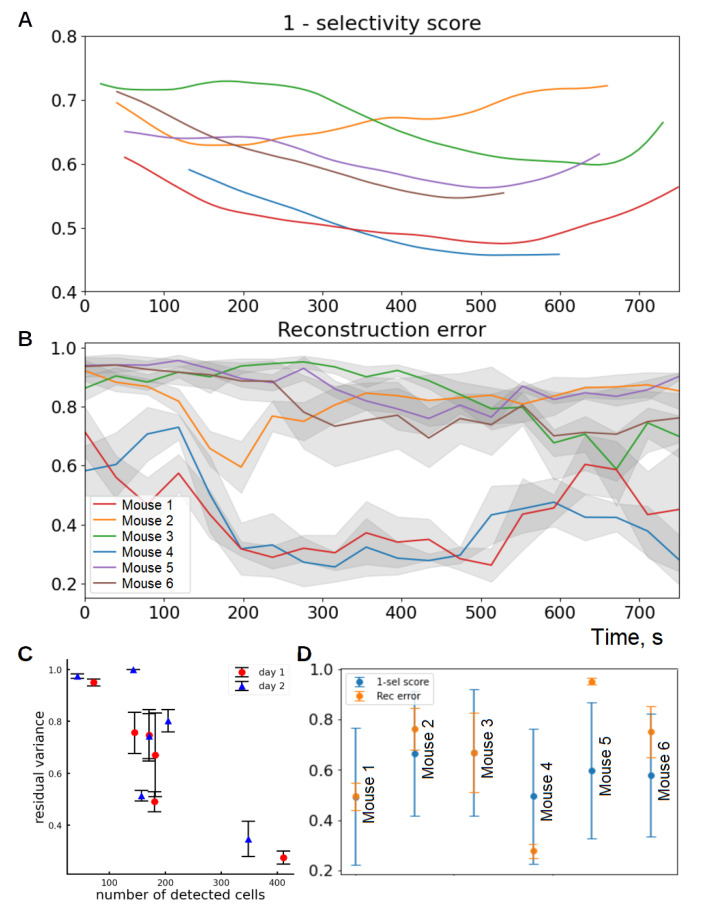
(**A**) Negated mean selectivity score plus one (unselectivity score), which was interpolated, smoothed with running average filter (window length 250 s) and plotted in the time domain. (**B**) Evolution of reconstruction error within the timeline of the 1st day session. Dimensionality reduction was performed for a sliding time window of length 250 s. Shadings represent standard deviations for LE with different graph construction parameters. NC stands for the number of cells registered. (**C**) Residual variance of the embedding depends on the number of detected cells. (**D**) Distribution of mean 1-selectivity score and of mean reconstruction error across different animals. These distributions demonstrate cosine similarity of 0.968 ± 0.047.

Next, we estimated the quality of the decoding of the space. The reconstruction error of the track space in the embedding (residual variance, RV) decreased with the number of registered cells (Figure 6A). We attribute this to the fact that nonlinear dimensionality reduction is able to distinguish “population” variables from the aggregate activity of many cells.

### 3.5. Representation Quality Dynamics over Time

The residual variance of track representation decreased over time as the mice became familiar with the environment explored (Figure 6 B). This effect was stronger the more cells were recorded for a given animal. We attribute this to the gradual formation of a population code in which information about the environment is distributed among many neurons. We compared these data with the negated average selectivity score for each mouse. The mousewise distributions of mean reconstruction error and of mean selectivity score demonstrate cosine similarity of 0.968 (Figure 6D). This result shows the consistency of different measures of quality of spatial coding.

## 4. Discussion

The main finding of this study is that place representations are promptly formed in mice during free exploration in a completely novel environment. This is consistent with the previous study by Muller et al. [32], where it was shown that place-selective cell firing began within a few minutes in rats exploring a novel environment. Moreover, recent data [8] show a similar distribution of place field forming times, nominated in laps, for mice navigating in a regular manner in a virtual environment. Here, we extend these results to a natural environment with completely free navigation conditions, where mice were capable of arbitrarily choosing the moving direction.

It is known that several types of spatial representation remapping may occur between different navigating sessions, including full, partial and rate remapping [33,34]. However, the retention of the cognitive maps was also observed [3,35]. The ratio of animals with preserved cognitive maps on the 2nd day in our experiments appeared to be consistent with the previous study [7], where it was shown that the global remapping is a stochastic process and several distinct cognitive maps can coexist in the same animal. Importantly, multiple visual cues, distal and proximal, were used in this study, which also is consistent with our experimental design. This could serve as an additional verifier that the mice trained in our paradigm demonstrated normal parameters of spatial representations even in the absence of reinforcement and goal-directed behavior.

The selectivity score dynamics that we observed suggest that the strength of place coding of the imaged CA1 population increases within each session, but without significant cumulation on further sessions. Nevertheless, we observed a reduction in average tuning latency on the second day, when the environment was familiar to the animals. This can be interpreted in the following manner: the strength of place coding starts from similar levels each session but increases faster in a familiar environment than in a novel one. Such dynamics can be associated with a gradual improvement in place coding between trials [36]. However, in this case, one could expect a more robust rise in the selectivity score in mice with retained representations or at least in place cells preserving their place fields, but we have not observed any significant difference. This implies that faster tuning of cognitive maps may be conditioned by some mechanisms not at the level of individual place cells but at the level of the whole CA1 population. Additional research should be done to clarify this question.

It is known that not only place cells may contribute to spatial coding [4,31,37]. By means of a population analysis, we demonstrated that a population of all registered cells as a whole can encode the space of the environment and that the quality of such encoding complements the average selectivity score. This can provide a basis for the estimation of the exact contribution of non-place cells to spatial code by excluding the activity of place cells from population activity, which will be a subject of further analysis.

We have not considered the direction specificity of place cells. It is known that, in one-dimensional tracks, there are direction-specific place fields and one cell can have place fields that are specific in different directions [38,39]. However, given the complete arbitrariness of the animals’ trajectories in our paradigm, it is difficult to take the direction into account due to unequal statistics of directions. Given this, we targeted our procedure of place cell detection to omnidirectional place cells, taking into account only continuous statistics of selective cell firing during sequential animals’ entrances in the place field in any direction.

One could expect some improvement in spatial code stability evoked by edge, border and object-specific cells [40]. There are no distinct edges and borders within our behavior paradigm, though some cells may be specifically tuned at distal and proximal visual cues around the circular track. However, the evaluation of the exact contribution of such putative cue-specific cells to spatial coding, as well as their precise identification, are obstructed by the overlap of their activity with the activity of “regular” place cells, since cues are integral parts of the environment. Additional modifications to the experimental setup will be required to isolate the contribution of cue-specific cells in further studies.

Due to the dispersion in the number of cells detected across different animals, we checked the consistency of our statistical comparison by excluding mice #8–9 with the lowest number of detected neurons (see Table A1 of Appendix A). As a result, the *p*-value of the factor day for the comparison of t_spec decreased from a value of *p* = 0.0488 to a value of *p* = 0.0436 (Figure 4C), and the *p*-values of the comparison of selectivity score between the first and the second visit to a field changed from values of *p* = 0.0261 and *p* = 0.0006 to values *p* = 0.0153 and *p* = 0.0003 for the first and the second session, respectively (Figure 4E). This change did not alter the statistical significance of the results, and therefore we retained mice #8 and 9 in the analysis.

According to [41,42], place codes can be modulated by the exploratory behavior of animals. We did not find any clear behavioral triggers or environmental cues that could activate place cell tuning. Given the relatively high (25%) fraction of immediate early-tuned (at the first visit to a field) place fields, special attention should be paid to the precise registration of all behavior parameters from the first seconds of the mouse’s entry into the environment. Nevertheless, at the level of discrete behavior acts, no behavior acts can be completed within such time, which poses a question about some internal states of the network activity leading to the rapid emergence of observed specializations.

Such mechanisms were suggested in studies by Dragoi and Tonegawa [43,44], which reported a possibility that some pre-existing representations supply immediate place coding in a novel environment. However, the contribution of such representations to overall place coding may be accomplished by hippocampal replays between consequent sessions, which may lead to the engagement of so-called "slowly-firing cells" of higher plasticity [45]. Taken together, these approaches may explain the further distinction and completion of novel and familiar environments.

Despite the clear geometric and physical meaning of the LE algorithm utilized in our study, this approach has several drawbacks, which are common for many manifold learning methods. In particular, this method does not scale well with increasing amounts of accumulated data, because its implementation relies on the spectral decomposition of the affinity matrix or related operators, such as the Laplacian or the transition matrix. In general, this leads to a computational complexity of O(n ${}^{a}$), 2.4 < a < 3, depending on the particular implementation. There is also no natural method for constructing a low-dimensional embedding for the neural activity vector, which was not represented in the initial data and, therefore, did not contribute to the formation of the graph. For this purpose, one has to construct special approximate nonlinear operators [46], which is not always possible. This limits the possibility of using the constructed embedding to analyze new data. To address this issue, we are working on new robust neural network-based methods for the dimensionality reduction of calcium signal data.

## 5. Conclusions

We have estimated the basic parameters of place cell selectivity within an imaging session at the first and second days of circular maze exploration. On the first day, the mean tuning latency of all place fields in all mice equaled 247 s. On average, place specialization was attained at the seventh visit of an animal to a place field, while 25.1% of place fields were established at their first attendance. On the second day, 3 of 8 mice demonstrated retention of their spatial representation, while 5 of 8 mice did not. In both cases, tuning latency on the second day was significantly lower than on the first day. On each day, the mean selectivity score significantly rose within the session. However, no cumulation was observed on the second day, and the initial and ultimate selectivity scores did not differ significantly between the first and the second day. Moreover, no difference in selectivity score or tuning latency dynamics was detected between the mice that had map retention or underwent remapping, neither at the level of individual cells nor at the level of average values.

Additionally, our nonlinear dimensionality reduction performed on CA1 neuronal activity data revealed the geometry of the environment explored by the mice. The reconstruction error for the six most informative mice on the first day of exploration corresponded to the negated mean selectivity score of these mice.

Taken together, these results reveal the fast emergence and tuning dynamics of place cell codes and demonstrate the applicability of novel calcium indicators NCaMP7 and FGCaMP7 for the light-controlled analysis of neural functions in behaving mice.

## Data Availability

The data that support the findings of this study and any custom written code are available from the corresponding author upon reasonable request.

## Supplementary Materials

The following supporting information can be downloaded at: https://www.mdpi.com/article/10.3390/ijms1010000/s1, Videos S1–S3: Samples of raw calcium signal captured with an NVista HD miniscope from different animals. Scale bars, 100 µm.

## Appendix A. Extended Data Tables and Figures

**Table A1:** Mousewise statistics for each registered session. 1PF Cells, single-field place cells. N spec and T spec, the average number of visit to a place field and correspondent time in seconds, since which this place field is considered tuned. First visit and Last visit sel.sc., the average values of selectivity score at the first and at the last attendance of a place field.

Mouse	Session	Calcium Sensor	Cells	Place Cells	Place Fields	1PF Cells	N Spec	T Spec	First Visit sel.sc.	Last Visit sel.sc.
Mouse 1	1	GCaMP6s	207	59	63	55	8.63	217.8	0.23	0.38
	2		235	24	28	21	8.79	219.2	0.10	0.30
	3		233	30	34	26	5.59	177.8	0.06	0.38
Mouse 2	1	GCaMP6s	263	90	101	79	10.10	237.3	0.20	0.30
	2		297	88	102	74	5.69	146.5	0.23	0.38
	3		228	80	92	68	7.37	196.4	0.19	0.41
Mouse 3	1	GCaMP6s	320	121	135	107	4.83	170.4	0.34	0.45
	2		315	106	114	98	2.21	152.2	0.41	0.44
	3		301	113	127	99	2.14	127.0	0.41	0.36
Mouse 4	1	GCaMP6s	562	257	298	218	3.90	256.4	0.32	0.49
	2		487	247	294	202	4.07	246.6	0.30	0.49
Mouse 5	1	NCaMP7	283	74	82	66	7.99	252.1	0.20	0.35
	2		317	86	96	77	6.58	186.0	0.09	0.46
Mouse 6	1	NCaMP7	200	26	30	22	6.63	245.1	0.15	0.33
	2		189	11	13	9	4.62	208.8	0.09	0.58
Mouse 7	1	GCaMP7f	292	130	174	87	11.26	305.6	0.16	0.31
	2		239	72	81	63	6.70	153.9	0.21	0.35
Mouse 8	1	FGCaMP7	95	10	10	10	6.10	144.4	0.31	0.31
	2		123	35	38	32	5.68	102.4	0.30	0.47
Mouse 9	1	NCaMP7	103	12	19	7	7.26	271.5	0.23	0.35

**Table A2:** Cell matching and remapping rate statistics between sessions. We considered that the cognitive map retained between sessions in case of the significant similarity of the distribution of place field location shifts between sessions to a normal distribution around zero (*p* < 0.05, Chi-square test).

Mouse	Sessions Compared	Cells Matched	Retention *p*-Value	Retention of Map
Mouse 1	1 vs. 2	157	0.886	no
	2 vs. 3	179	0.657	no
	1 vs. 3	153	0.839	no
Mouse 2	1 vs. 2	127	0.839	no
	2 vs. 3	138	0.552	no
	1 vs. 3	116	0.006	yes
Mouse 3	1 vs. 2	229	<0.001	yes
	2 vs. 3	217	<0.001	yes
	1 vs. 3	116	<0.001	yes
Mouse 4	1 vs. 2	396	<0.001	yes
Mouse 5	1 vs. 2	208	<0.001	yes
Mouse 6	1 vs. 2	120	0.456	no
Mouse 7	1 vs. 2	69	0.457	no
Mouse 8	1 vs. 2	77	0.237	no
